# The Functional Polymorphisms of miR-146a Are Associated with Susceptibility to Severe Sepsis in the Chinese Population

**DOI:** 10.1155/2014/916202

**Published:** 2014-02-20

**Authors:** Yiming Shao, Jia Li, Yujie Cai, Yuliu Xie, Guoda Ma, You Li, Yanyan Chen, Gen Liu, Bin Zhao, Lili Cui, Keshen Li

**Affiliations:** ^1^Guangdong Key Laboratory of Age-Related Cardiac and Cerebral Diseases, Affiliated Hospital of Guangdong Medical College, 57 Renmin Avenue, Xiashan District, Zhanjiang 524001, China; ^2^Intensive Care Unit, Affiliated Hospital of Guangdong Medical College, Zhanjiang 524001, China

## Abstract

MicroRNA-146a (miR-146a) acts as a pivotal regulatory molecule in immune response and various diseases, such as carcinoma and autoimmune diseases. Growing evidences have demonstrated the association of miR-146a gene single-nucleotide polymorphisms (SNPs) with risk of several diseases, but no genetic relevance studies of miR-146a gene polymorphisms to sepsis have been reported by now. Our study has analyzed the association of sepsis with two functional miR-146a gene SNPs rs2910164 G/C and rs57095329 A/G in a Chinese Han population (226 sepsis cases; 206 healthy controls). Our results indicated a higher prevalence of the miR-146a gene SNP rs2910164 C allele and CC genotype in patients with severe sepsis (rs2910164G versus rs2910164C: *P* = 0.0029, odds ratio (OR) = 1.664; GG+GC versus CC: *P* = 0.0045, OR = 1.947). Neither the genotype nor the allele in rs57095329 showed significant differences between the septic cases and the controls (*P* = 0.5901 and 0.3580, resp.), and no significant difference was observed in the subgroups. In addition, we confirmed that the two SNPs rs2910164 and rs57095329 could functionally affect the miR-146a expression levels and the reduction of miR146a was accompanied with the upregulation of the expression levels of TRAF-6 and IRAK-1 in severe sepsis patients. This present study might provide valuable clinical evidence that miR-146a gene polymorphism rs2910164 is associated with the risk of severe sepsis.

## 1. Introduction

Sepsis is a systemic disease characterized by microbial infection and systemic inflammatory response syndrome (SIRS), which has high morbidity and mortality rates in Intensive Care Units (ICUs) [1]. The exacerbation of sepsis is always accompanied by the dysfunction of the innate and adaptive immune responses [2, 3] and followed by severe sepsis, septic shock, and multiple organ dysfunction syndrome (MODS) [4]. Although the pathophysiology of sepsis is not thoroughly understood, genetic epidemiologic studies suggest a strong genetic influence on the pathogenesis of sepsis [5]. To date, a number of evidences have demonstrated that functional genetic variants within genes involved in the innate and adaptive immune responses play a pivotal role in a patient's predisposition to sepsis and their prognosis [6–13].

MicroRNAs (miRNAs) are a type of small, noncoding, single-stranded RNAs that can posttranscriptionally downregulate gene expression by binding to the 3′ untranslated region (3′UTR) of target mRNAs [14]. Many studies have identified miRNAs as vital regulatory molecules that may be involved in the pathogenesis of immune and inflammatory pathologies in human diseases [15]. One of these conserved miRNAs is miR-146a, which is well known for its important regulation of the immune response and inflammation [16]. miR-146a is induced upon the activation of toll-like receptor 4 (TLR4) in the NF-*κ*B-dependent signaling pathway, leading to the downregulation of IL-1 receptor-associated kinase 1 (IRAK1) and TNF receptor-associated factor 6 (TRAF6) [17]. Recent studies have demonstrated that the serum or plasma levels of miR-146a in septic patients were significantly decreased compared to those of normal controls and SIRS patients [18, 19], suggesting that miR-146a may be significantly associated with sepsis.

Currently, many studies have shown that two functional single nucleotide polymorphisms (SNPs), rs2910164 G/C and rs57095329 A/G, in the miR-146a gene may influence the expression level of mature miR-146a and can result in genetic predisposition to diseases [20–22]. The rs2910164 G/C SNP, which is located in the stem region of precursor miR-146a and results in a C:U miss-pair instead of a G:U pair [23], affects the integrity of the stem region of pre-miR-146a, as well as the processing of pre-miR-146a into mature miR-146a [23]. This polymorphism has been reported to contribute to the susceptibility for several diseases, such as papillary thyroid carcinoma [20], prostate cancer [24], and hepatocellular carcinoma [25]. For the other polymorphism in our study, the rs57095329 A/G SNP in the miR-146a promoter region, it has also been shown to reduce the level of mature miR-146a by decreasing the binding of its transcription factor V-Ets oncogene homolog 1 (Ets-1) and was associated with the risk of developing systemic lupus erythematosus (SLE) [22]. However, currently there are no reports regarding the genetic association of these two SNPs in miR-146a and the risk of sepsis.

In the present study, a case-control study was carried out to determine whether there is an association between the two polymorphisms within the miR-146a gene and sepsis in a Chinese population. Moreover, the expression levels of IRAK1, TRAF6, and miR-146a were also determined in the severe sepsis and healthy subjects to analyze whether these polymorphisms are associated with the expression levels of these cytokines.

## 2. Materials and Methods

### 2.1. Participant Recruitment

A total of 226 septic patients of a Chinese population were recruited from the ICU department of the Affiliated Hospital of Guangdong Medical College between April 2011 and June 2013. Their blood samples were collected upon the diagnosis of sepsis, severe sepsis, or septic shock, which was established according to the International Sepsis Definitions Conference [1, 4]. Patients that were younger than 18 years old and patients who suffered from diabetes, autoimmune diseases, malignancies, human immunodeficiency virus (HIV), or acquired immune deficiency syndrome (AIDS) or receiving immunosuppressive, steroid, or radiation therapy were excluded from this study. The following clinical parameters were recorded for each patient: age, sex, dysfunctional organs, source of infection, blood microbiological cultures, and Acute Physiology and Chronic Health Evaluation (APACHE) II score [26]. A total of 206 age-, race-, and sex-matched controls were enrolled from the Health Examination Center at this hospital. Participants in the healthy control group were free from autoimmune diseases, hypertension, diabetes, cancer, and major cardiac, renal, hepatic, and endocrinological disorders. Written informed consent was obtained from the participants prior to their enrollment in the study. Each patient's capacity to consent was confirmed by a family member when necessary. This study was approved by the Ethics Committee of the Affiliated Hospital of Guangdong Medical College, China.

### 2.2. DNA Extraction and Genotyping

Genomic DNA was extracted from whole blood samples from all of the patients and controls using the TIANamp Blood DNA Kit (Tiangen Biotech, Beijing, China) according to the manufacturer's instructions and was stored at −80°C before genotyping. A total of 432 individuals were genotyped for the two SNPs (rs2910164 G/C and rs57095329 A/G) using the SNaPshot technique (Applied Biosystems, Foster City, CA, USA). The PCR primers used for the polymorphic site rs2910164 were 5-GAACTGAATTCCATGGGTTG-3 and 5-CACGATGACAGAGATATCCC-3, and the primers used for rs57095329 were 5-TCATTGGGCAGCCGATAAAG-3 and 5-AGGAAGTTCTGGTCAGGCG-3. Genotyping was conducted by polymerase chain reaction (PCR). The 10-*μ*L multiplex SBE reactions included 5 *μ*L of SNaPshot Multiplex Kit (ABI), 2 *μ*L of template DNA containing the multiplex PCR products, 2 *μ*L of ddH_2_O, and 1 *μ*L of primer mix. The PCR reactions were carried out using the following cycling parameters: 96°C for 1 min (the initial denaturation), followed by 28 cycles of 96°C for 10 seconds (denaturation), 52°C for 5 seconds (annealing), and 60°C for 30 seconds (extension). The products were further purified by incubation with 1 U of shrimp alkaline phosphatase (Promega) at 37°C for 1 h, followed by incubation at 75°C for 15 min. Then, 0.5 *μ*L of the purified product was added to 0.5 *μ*L of the Lizl20 Size Standard and 9 *μ*L of Hi-Di formamide, followed by incubation at 95°C for 5 min. The samples were then added into the ABI Prism 3130XL genetic sequence analyzer. The final data were analyzed using GeneMapper 4.1 (Applied Biosystems, Foster City, CA, USA).

### 2.3. Mononuclear Cells Isolation

In total, 37 cases from 127 patients with severe sepsis and 40 healthy controls form 206 healthy subjects that were chosen at random for the isolation of mononuclear cells. The peripheral blood mononuclear cells (PBMCs) using density gradient centrifugation method with LymphoprepTM (Axis-Shield PoCAS, Oslo, Norway) were isolated as soon as possible when the blood samples were collected. In brief, blood samples were mixed with equal volume of 0.9% NaCl, and then the diluted blood was slowly added to tubes containing a Ficoll premium solution to make the blood layered upon the Ficoll. Samples were centrifuged at 800 ×g for 30 min at room temperature. After centrifugation, the mononuclear cells form a distinct band at the medium interface. The cells were then shifted to other tubes using Pasteur pipette without removing the upper layer and washed with 0.9% NaCl. Then, samples were centrifuged again at 250 ×g for 10 min. The mononuclear cells were harvested and stored at −80°C.

### 2.4. RNA Extraction, Reverse Transcription, and Real-Time PCR

RNA was extracted from PBMCs of the 37 cases and 40 controls immediately after the isolation of mononuclear cells using the UNIQ-10 Column TRizol Total RNA Extraction Kit (Sangon Biotech, Shanghai, China) as per the manufacturer's instructions. The integrity of the RNA was checked using 1% agarose gel electrophoresis. The RNA was reverse transcribed using the First Strand cDNA Synthesis Kit (Thermo) as per the manufacturer's instructions. The expression levels of IRAK-1 and TRAF-6 were analyzed by quantitative real-time PCR with the SYBR green method. The IRAK-1 and TRAF-6 expression levels were analyzed in triplicate, and expression was normalized to the level of glyceraldehyde 3-phosphate dehydrogenase (GAPDH), which was used as an internal control. IRAK-1, TRAF-6, and GAPDH specific primers were diluted to a final concentration of 1 *μ*M. The 10-*μ*L PCR reactions consisted of 5 *μ*L of SYBR Green PCR master mix (TaKaRa), 2 *μ*L of each specific forward and reverse primer, and 1 *μ*L of template cDNA. The RT-PCR reactions were then conducted in a LightCycler_480 sequence detector system (Roche Applied Sciences) and were incubated at 95°C for 30 seconds, followed by 40 cycles of 95°C for 5 seconds and 62°C (for IRAK-1 only) or 61°C (for TRAF-6 only) for 20 seconds. The expression levels of IRAK-1 and TRAF-6 were calculated using the 2^ΔDDCt^ method.

### 2.5. miRNA Extraction, Reverse Transcription, and Real-Time PCR

miRNA was also extracted from PBMCs of the same 37 cases and 40 controls using the miRcute miRNA Isolation Kit (Tiangen Biotech, Beijing, China) according to the manufacturer's instructions. miR-146a and U6 were reverse transcribed using the following protocol immediately. The 20 *μ*L reactions contained 8 *μ*L of miRNA, 4 *μ*L of 5x buffer, 3 *μ*L of RNase free H_2_O, 2 *μ*L of 10 mM dNTP mix, 1 *μ*L of RNase inhibitor, 1 *μ*L of RTase, and 1 *μ*L of the miR-146a or U6 RT primer (final concentration of 1 *μ*M). The reactions were then incubated at 16°C for 30 min, followed by 42°C for 45 min and, finally, 85°C for 10 min. The expression levels of miR-146a were analyzed by qRT-PCR using the SYBR green method. All miR-146a samples were analyzed in triplicate and the expression was normalized to the level of U6, which was used as an internal control. Forward and reverse primers for miR-146a and U6 were diluted to a final concentration of 1 *μ*M. The 10-*μ*L PCR reactions consisted of 5 *μ*L of SYBR Green PCR master mix (TaKaRa), 1 *μ*L of each specific forward and reverse primer, 2 *μ*L of ddH_2_O, and 1 *μ*L miR-146a or U6 cDNA. QRT-PCR was then performed in a LightCycler_480 sequence detector system (Roche Applied Sciences), and the samples were incubated at 95°C for 30 seconds, followed by 40 cycles of 95°C for 5 seconds and 62°C for 20 seconds. The miR-146a expression levels were obtained using the 2^ΔDDCt^ method.

### 2.6. Statistical Analyses

Statistical analyses were conducted using SPSS version 19.0 (IBM, NY, USA) and GraphPad Prism 4.0 (GraphPad Software Inc., San Diego, CA, USA). Genotype and allele frequencies between the cases and controls were calculated using Chi-squared test or Fisher's exact test. Deviation of the genotype or allele frequency was assessed using Hardy-Weinberg equilibrium (HWE). Power analysis was performed using the software QUANTO 1.2 (http://hydra.usc.edu/gxe/). The comparisons of the IRAK-1, TRAF-6, and miR-146a expression levels between the patients and controls were evaluated using Student's *t*-test for the normally distributed data or a Mann-Whitney *U* test for the nonparametric data. An ANOVA was performed for all other calculations. The false discovery rate for multiple hypotheses testing was calculated using the Benjamin-Hochberg (BH) multiple testing corrections. Our data are expressed as the mean ± SEM or as percentage frequencies. Statistical significance was defined as a *P* value < 0.05.

## 3. Results

### 3.1. Clinical Characteristics

The clinical parameters of 226 patients with sepsis and 206 healthy controls were shown in Table 1. There were no significant differences in the age and gender distributions between the sepsis and healthy control groups. The respiratory tract (80.1%), primary blood stream (39.8%), and abdomen (37.2%) were the main sites of infection. Gram-negative infection (36.3%) and mixed infection (34.5%) were the primary infection types, while fungal infection accounted for 20.8%. Two or more organ dysfunctions existed in 68.6% of the total patients. Severe sepsis accounted for 56.2% of the sepsis patients. The 28-day mortality rate was 42.9% in this study cohort.

### 3.2. Genotype and Allele Frequency Distributions among Sepsis Patients and Controls

In total, 226 sepsis patients and 206 healthy individuals were successfully analyzed for the rs2910164 SNP and 222 patients and 205 controls were genotyped for the rs57095329 SNP. The genotype and allele frequency distributions of the two SNPs in the cases and controls are listed in Table 2. The distributions of the genotype frequencies for the two SNPs complied with the Hardy-Weinberg equilibrium in both the cases and controls (all *P* > 0.05, data not shown). Our results showed a significant difference between the sepsis patients and the healthy controls concerning the genotype frequency of rs2910164 (*P* = 0.047). According to a dominant model (GG/GC versus CC), a significant difference was found in the sepsis cases compared to the healthy controls (*P* = 0.035, OR = 1.508). The G allele frequency of the rs2910164 SNP in the sepsis cases was significantly higher than that in healthy controls (*P* = 0.019, OR = 1.408). Nevertheless, after Benjamini-Hochberg (BH) multiple testing corrections, no statistically significant differences were observed between the sepsis cases and controls (genotype frequencies *P (corr) = 0.063; GG/GC versus CC *P (corr) = 0.063; allele frequencies *P (corr) = 0.063). With regards to the rs57095329 polymorphism, no statistically significant difference was observed between the sepsis patients and the healthy controls for both the genotype and allele frequencies of the rs57095329 SNP (genotype frequencies *P* = 0.818; allele frequencies *P* = 0.694). Moreover, power analysis showed that with our sample size, we would have 95% power for rs2910164 and 87% power for rs57095329 to detect a genotype relative risk with an odds ratio of 1.5 at a significance level of 0.05.

### 3.3. Genotype and Allele Frequency Distributions among Sepsis Subtypes

All sepsis cases were divided into three subtypes (sepsis, septic shock, and severe sepsis) to investigate the association of the two SNPs with the sepsis subtypes. As presented in Table 3, there were statistically significant differences in the genotype and allele frequencies of rs2910164 between the severe sepsis subtype and the healthy controls. The frequency of the G allele of rs2910164 in the severe sepsis subtype was significantly higher than that of the healthy controls (*P* = 0.0029, OR = 1.664, 95% CI: 1.196–2.316 for allele). In a dominant model (GG/GC versus CC), a significant difference was found in the severe sepsis subtype compared to the healthy controls (*P* = 0.0045, OR = 1.947, 95% CI: 1.229–3.083 for genotype). Furthermore, after BH multiple testing corrections, the significant difference still remained [P*(corr) = 0.0045 for genotype; P*(corr) = 0.0045 for allele]. There was no statistically significant difference observed between the sepsis or septic shock subtypes and the healthy controls. We also separated the sepsis cases into two subtypes on the basis of the patients' 28-day mortality. A statistically significant difference in the genotype frequency of rs57095329 was found between the survivors and the nonsurvivors among all sepsis patients (*P* = 0.015, P*(corr) = 0.030) but no significant difference was observed in allele frequency of rs57095329 (Table 4). Moreover, we did not observe an association between the rs2910164 SNP and the 28-day mortality rate in the sepsis patients.

### 3.4. Expression Levels of miR-146a, TRAF-6, and IRAK-1

**Figure 1:**
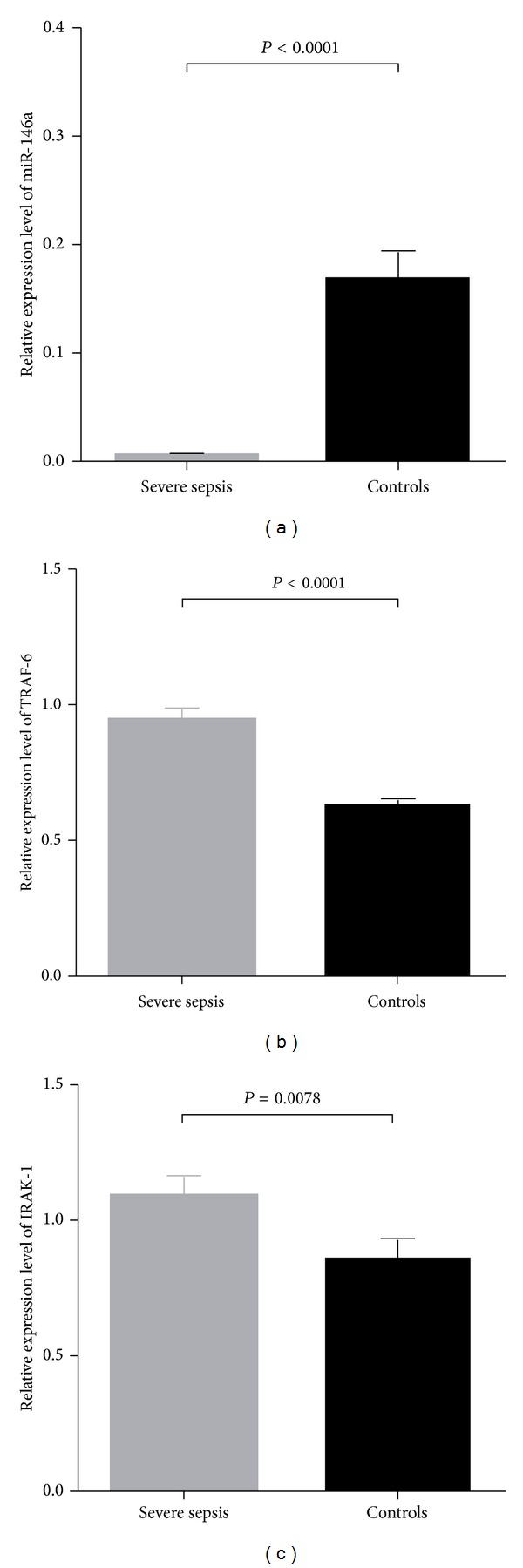
Expression levels of miR-146a (a), TRAF-6 (b), and IRAK-1 (c) in severe sepsis patients (*n* = 37) and normal controls (*n* = 40), respectively.

In total, 37 patients with severe sepsis and 40 healthy individuals were stochastically chosen to investigate the expression levels of miR-146a, TRAF-6, and IRAK-1 in PBMCs. Our results revealed that the relative expression levels of miR-146a in patients with severe sepsis were significantly lower than those in the healthy controls (*P* < 0.0001) (Figure 1(a)). The relative expression levels of TRAF-6 and IRAK-1 were significantly higher in patients with severe sepsis compared to those of the healthy controls (*P* < 0.0001 and *P* = 0.0078, resp.) (Figures 1(b) and 1(c)).

### 3.5. Influence of the miR-146a Polymorphisms on the Expression Levels of miR-146a, TRAF-6, and IRAK-1

**Figure 2:**
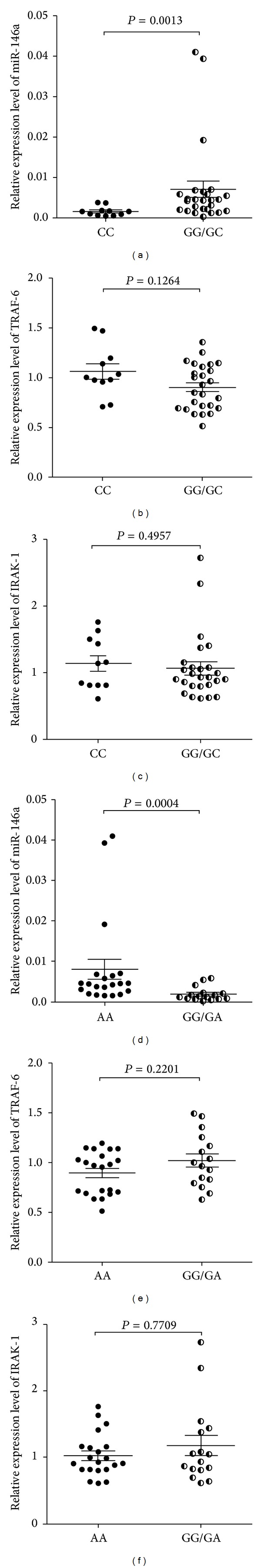
The distribution of miR-146a (a), TRAF-6 (b), and IRAK-1 (c) expression levels in groups of severe sepsis patients with different rs2910164 genotypes. The distribution of miR-146a (d), TRAF-6 (e), and IRAK-1 (f) expression levels between groups of severe sepsis with different rs57095329 genotypes. The horizontal line stands for the median expression level with each group.

We further explored the influence of the two polymorphisms on the expression levels of miR-146a, TRAF-6, and IRAK-1. As shown in Figure 2, significant differences in the expression levels of miR-146a were observed in the different rs2910164 and rs57095329 genotypes in severe sepsis subgroup. The expression levels of miR-146a in the rs2910164 CC patients were significantly lower than those in the GG/GC patients (*P* = 0.0013) and the expression levels of miR-146a in the rs57095329 AA patients were significantly higher than those in the GG/GA patients (*P* = 0.0004). However, no significant differences in TRAF-6 expression levels were observed between the severe sepsis subgroup with the different rs2910164 and rs57095329 genotypes (*P* = 0.1264 for rs2910164 SNP and *P* = 0.2201 for rs57095329 SNP, resp.). Additionally, no significant associations were observed between the two SNPs and the expression levels of IRAK-1 (*P* = 0.4957 for rs2910164 SNP and *P* = 0.7709 for rs57095329 SNP, resp.).

## 4. Discussion

To the best of our knowledge, the present study was the first to investigate the relevance of two miRNA-146a gene polymorphisms rs2910164 and rs57095329 to the risk of sepsis in a Chinese population. Our data demonstrated that patients carrying the rs2910164 C allele of miRNA-146a had suffered from a lower risk of severe sepsis. Severe sepsis patients with the CC genotype of rs2910164 expressed a lower level of miRNA-146a compared with the GG/GC genotypes. No significant association was observed between the rs57095329 SNP and the risk of sepsis. Moreover, we identified a statistically significant difference concerning the genotype frequency of rs57095329 polymorphism between the survivors and nonsurvivors of all sepsis patients, but not the allele frequency. It seemed that AA genotype acted as a protective factor in the survivors of sepsis. However, as most data related to the association between SNP rs57095329 and sepsis were negative and this association was only observed in genotype frequency, this association still needs to be confirmed in larger and multiple populations.

Our study demonstrated that severe sepsis patients expressed lower levels of miR-146a and higher levels of TRAF-6 and IRAK-1 than the healthy controls. This observation is consistent with the previous findings that the level of miR-146a in PBMCs was significantly lower in sepsis patients than in SIRS patients and normal controls [18, 19]. Early in the process of sepsis, the innate immune system of the host is activated in response to the infection [27]. Upon activation, immunocytes express the pattern recognition receptors (PRRs), including TLRs, through which signaling pathways can be recognized and transduced [2]. TRAF-6 and IRAK-1 are two pivotal adapter molecules that are downstream of the TLR signaling pathway [28]. Under the stimulation of TLR4, the cascade of TRAF-6 and IRAK-1 results in the activation of the NF-*κ*B signaling pathway [28] and then activated NF-*κ*B induces the transcription of miR-146a [29], and the mature miR-146a in turn inhibits the expression of TRAF-6 and IRAK-1 thus attenuating the TLR signaling pathway [17]. Our results support this view and show that deregulated miR-146a negatively regulated its target molecules TRAF-6 and IRAK-1 in severe sepsis patients.

Recently, two SNPs in miRNA-146a, rs2910164, and rs57095329 were reported that they could influence the expression level of mature miR-146a and thus affect an individual's susceptibility to various diseases [20–22, 24, 25, 30]. The functional significance of the rs2910164 SNP was experimentally identified by Jazdzewski et al. [20]. This rs2910164 G/C polymorphism is within the passenger strand of the pre-miR-146a sequence; the C allele was found to attenuate the processing of pri-miR-146a, decreasing the amount of mature miR-146a and increasing the expression levels of its target genes, including TRAF-6 and IRAK-1 [20]. Jazdzewski et al. further showed that the additional mature miR-146a*G and miR-146a*C from the passenger strand of the pre-miR-146a regulated genetic processing [30]. With regards to the rs57095329 polymorphism, Luo et al. reported that this polymorphism was located in the promoter of miR-146a and the risk-linked G allele reduced the protein-binding affinity of the transcription factor Ets-1, thus, resulting in decreased levels of miR-146a in patients with SLE [22]. Additionally, the rs2910164 and rs57095329 SNPs were both reported to be common polymorphisms in the Chinese population [21, 22]. So these two functional SNPs of miR146a were chosen for this case-control study to ascertain whether these two SNPs could affect an individual's susceptibility to sepsis.

Considering the functional importance of the rs2910164 and rs57095329 SNPs, we further investigated the association of the two SNPs with the expression levels of miR-146a and key adapter molecules downstream of TRAF-6 and IRAK-1. Our data revealed that the GG/GC genotypes of rs2910164 showed a higher expression level of mature miR-146a, which was in accordance with the previous investigations reported in healthy individuals in the Chinese population [21] and in hepatocellular carcinoma patients in the Chinese population [25]. The higher expression levels of mature miR-146a in patients with the rs2910164 GG/GC genotypes appeared to be relevant to the hyper inflammatory response in severe sepsis. The rs57095329 GG/GA genotypes tended to downregulate the expression level of mature miR-146a, which was consistent with results reported in healthy individuals in the Chinese population [22].

Many studies have reported that miR-146a can negatively regulate the expression levels of TRAF-6 and IRAK-1 in different cell lines and diseases [31–35]. Our study showed similar results; the downregulation of miR-146a expression was accompanied by the upregulation of TRAF-6 and IRAK-1 in severe sepsis patients. Moreover, we demonstrated that the two functional SNPs rs2910164 and rs57095329 within the miR-146a gene could affect the expression level of mature miR-146a, which is consistent with the previous studies mentioned above [21, 22, 25]. However, when we attempted to investigate the genetic association between the genotype frequency distribution of the two miR-146a polymorphisms and the expression levels of TRAF-6 and IRAK-1, we failed to observe any significant differences. We determined that there could be three reasons for these negative results. First, the expression levels of TRAF-6 and IRAK-1 are not regulated only by miR-146a in the immune system of the host, the impact in miR-146a expression caused by the miR-146a SNPs might be limited, and our experiment is not sensitive enough to observe the impact on the expression levels of TRAF-6 and IRAK-1. Second, as the general pathogenic conditions of sepsis patients in clinical practice, it is difficult to achieve homogeneity. For instance, various drug treatments for each patient may have an impact, despite the fact that we have excluded patients who were being treated with immunosuppressive, steroid, or radiation therapy in our study. Therefore, the expression of genes in the whole inflammatory-associated signaling pathway might also be influenced. Last, the sample size of the case and control groups in this study cohort was relatively not large, and the group we recruited in our study was only from the west Guangdong Province in China with geographical and population restrictions; the further study on this association using larger cohorts and multiple populations is needed. Nevertheless, our results confirmed that the expression level of miR-146a was significantly lower in severe sepsis patients and was accompanied by higher expression levels of TRAF-6 and IRAK-1 compared to the healthy controls.

## 5. Conclusions

Our present study demonstrated a significant association of the rs2910164 SNP in the pre-miR-146a gene with the susceptibility of a patient to severe sepsis in a Chinese population. Moreover, the rs2910164 and rs57095329 SNPs could functionally affect the miR-146a expression levels and the miR-146a could negatively regulate the expression of IRAK1 and TRAF6 in severe sepsis patients. We proposed that the rs2910164 polymorphism of miR-146a could influence the susceptibility of an individual to severe sepsis and might be used as a potential biomarker for diagnosing severe sepsis in clinical practice.

## Figures and Tables

**Table 1:** Clinical characteristics of sepsis cases and healthy controls.

Characteristics	Cases (*n* = 226)	Controls (*n* = 206)	*P* value
	*N* (%)	*N* (%)	
Age (years)	61.61 ± 15.59	59.87 ± 10.66	0.174
Male/female, *n*	153/73	140/66	0.954
Organ dysfunction			
One, *n* (%)	37 (16.4)	N.A	
Two, *n* (%)	72 (31.9)	N.A	
Three or above, *n* (%)	83 (36.7)	N.A	
Sepsis status			
Sepsis, *n* (%)	34 (15.0)	N.A	
Septic shock, *n* (%)	65 (28.8)	N.A	
Severe sepsis, *n* (%)	127 (56.2)	N.A	
Source of infection, *n* (%)			
Respiratory tract infection	181 (80.1)	N.A	
Primary bloodstream infection	90 (39.8)	N.A	
Wound infection	23 (10.2)	N.A	
Abdominal infection	84 (37.2)	N.A	
Urinary tract infection	7 (3.1)	N.A	
Catheter-associated infection	31 (13.7)	N.A	
Others	22 (9.7)	N.A	
Pathogens, *n* (%) (positive blood cultures)			
Gram-negative	82 (36.3)	N.A	
Gram-positive	40 (17.7)	N.A	
Mixed Gram-negative and -positive	78 (34.5)	N.A	
Fungus	47 (20.8)	N.A	
Negative blood cultures	12 (5.3)	N.A	
APACHE II score	20.5 ± 5.9	N.A	
28-day mortality, *n* (%)	97 (42.9)	N.A	

N.A: not applicable; APACHE II: Acute Physiology and Chronic Health Evaluation II.

**Table 2:** Genotype and allele frequencies distribution in patients with sepsis and controls.

Genotype	All sepsis cases, *N* (%)	Controls, *N* (%)	*P* value	*P* value*	OR (95% CI)
rs2910164					
Total	**226**	**206**	**0.047**	**0.063**	
CC	88 (38.94)	101 (49.03)			
GC	114 (50.44)	93 (45.15)			
GG	24 (10.62)	12 (5.82)			
GG/GC	138 (61.06)	105 (50.97)	0.035	0.063	1.508 (1.029–2.211)
GC/CC	202 (89.38)	194 (94.18)	0.072	0.072	0.521 (0.253–1.070)
Allele					
C allele	290 (64.16)	295 (71.60)			1.000 (reference)
G allele	162 (35.84)	117 (28.40)	0.019	0.063	1.408 (1.056–1.878)

rs57095329					
Total	**222**	**205**	**0.818**	**0.830**	
AA	144 (64.86)	135 (65.85)			
GA	69 (31.08)	64 (31.22)			
GG	9 (4.05)	6 (2.93)			
GG/GA	78 (35.14)	70 (34.15)	0.830	0.830	1.045 (0.701–1.557)
GA/AA	213 (95.95)	199 (97.07)	0.527	0.830	0.714 (0.249–2.042)
Allele					
A allele	357 (80.41)	334 (81.46)			1.000 (reference)
G allele	87 (19.59)	76 (18.54)	0.694	0.830	1.071 (0.761–1.508)

OR: odds ratio; 95% CI: 95% confidence interval; *false discovery rate-adjusted *P* value for multiple hypotheses testing using the Benjamini-Hochberg method.

**Table 3:** Genotype and allele frequencies distribution in different sepsis status and healthy controls.

Genotype	Healthy control *N* (%)	Sepsis (subtype) *N* (%)	Septic shock *N* (%)	Severe sepsis *N* (%)	*P* value			*P* value*		
					*P*_1_	*P*_2_	*P*_3_	*P*_1_*	*P*_2_*	*P*_3_*
rs2910164										
Total	**206**	**34**	**65**	**127**	**0.712**	**0.888**	**0.0045**	**0.712**	**0.888**	**0.0045**
CC	101 (49.03)	15 (44.12)	31 (47.69)	42 (33.07)						
GG/GC	105 (50.97)	19 (55.88)	34 (52.31)	85 (66.93)						
Allele										
C	295 (71.60)	46 (67.65)	91 (70.00)	153 (60.24)						
G	117 (28.40)	22 (32.35)	39 (30.00)	101 (39.76)	0.564	0.740	0.0029	0.712	0.888	0.0045

rs57095329										
Total	**205**	**33**	**62**	**127**	**0.560**	**0.278**	**0.413**	**0.613**	**0.503**	**0.421**
AA	135 (65.85)	20 (60.61)	46 (74.19)	78 (61.42)						
GG/GA	70 (34.15)	13 (39.39)	16 (25.81)	49 (38.58)						
Allele										
A	334 (81.46)	52 (78.79)	105 (84.68)	200 (78.74)						
G	76 (18.54)	14 (21.21)	19 (15.32)	54 (21.26)	0.613	0.503	0.421	0.613	0.503	0.421

OR: odds ratio; 95% CI: 95% confidence interval. *False discovery rate-adjusted *P* value for multiple hypotheses testing using the Benjamini-Hochberg method. *P*_1_ and *P*_1_*: healthy control group versus sepsis group. *P*_2_ and *P*_2_*: healthy control group versus septic shock group. *P*_3_ and *P*_3_*: healthy control group versus severe sepsis group. Fisher's exact test *P*_3_ = 0.0045, OR = 1.947, 95% CI (1.229–3.083) for genotype in rs2910164; *P*_3_ = 0.0029, OR = 1.664, 95% CI (1.196–2.316) for allele in rs2910164.

**Table 4:** Genotype and allele frequencies distribution in surviving and nonsurviving patients.

Genotype	Survivors *N* (%)	Nonsurvivors *N* (%)	*P* value	*P* value*	OR (95% CI)
rs2910164					
Total	**129**	**97**	**0.148**	**0.296**	
CC	48 (37.21)	40 (41.24)			
GC	71 (55.04)	43 (44.33)			
GG	10 (7.75)	14 (14.43)			
Allele					
C	167 (64.73)	123 (63.40)			1.000 (reference)
G	91 (35.27)	71 (36.60)	0.771	0.771	0.944 (0.640–1.392)

rs57095329					
Total	**125**	**97**	**0.015**	**0.030**	
AA	90 (72.00)	54 (55.67)			
GA	29 (23.20)	40 (41.23)			
GG	6 (4.80)	3 (3.10)			
Allele					
A	209 (83.60)	148 (76.29)			1.000 (reference)
G	41 (16.40)	46 (23.71)	0.054	0.054	0.631 (0.394–1.011)

OR: odds ratio; 95% CI: 95% confidence interval; *False discovery rate-adjusted *P* value for multiple hypotheses testing using the Benjamini-Hochberg method.
